# 组氨酸激酶和组氨酸磷酸酶在肿瘤中的作用

**DOI:** 10.3779/j.issn.1009-3419.2021.102.28

**Published:** 2021-09-20

**Authors:** 娅芳 董, 慧敏 韩, 亚峰 李, 丽丽 郭

**Affiliations:** 030000 太原，山西医科大学附属人民医院，山西省精准医学中心，肾脏病山西省重点实验室 Key Laboratory of Kidney Disease, precision Medicine Center, The Shanxi Provincial People's Hospital, Shanxi Medical University, Taiyuan 030000, China

**Keywords:** 组氨酸激酶, 组氨酸磷酸酶, 肿瘤, 肺肿瘤, Histidine kinase, Histidine phosphatase, Cancer, Lung neoplasms

## Abstract

蛋白磷酸化修饰是最常见、最重要的蛋白质翻译后修饰方式。磷酸化修饰在细胞的增殖、分化、发育和代谢等生物学过程中发挥了重要的调控功能，与肿瘤的发生和发展也密切相关。蛋白激酶和磷酸酶对蛋白磷酸化修饰具有普遍的开/关调控作用。真核生物的蛋白磷酸化主要发生在丝氨酸、苏氨酸和酪氨酸残基，他们在肿瘤发生和发展中的作用已经得到了广泛的研究。但关于组氨酸磷酸化的研究受限于质谱分析和富集技术的发展研究较少。近年来，随着相关技术的快速发展和新的组氨酸磷酸酶的发现，使得研究人员越来越多关注到组氨酸磷酸化在肿瘤中的作用。因此，本文旨在对组氨酸磷酸化调控相关的组氨酸激酶和组氨酸磷酸酶在肿瘤中的作用作一综述。

## Abstract (en)

Phosphorylation is the most common and important post-translational modification of proteins, which plays an important role in the regulation of cell proliferation, differentiation, development and metabolism, and is closely related to the tumorigenesis and metastasis of cancer. Protein kinases and phosphatases generally regulate protein phosphorylation levels as a pair of opposite acting enzymes. Protein phosphorylation in eukaryotes occurs mainly in serine, threonine, and tyrosine residues, and their roles in tumorigenesis and development have been extensively studied. But the roles on histidine phosphorylation is less known due to the immature mass spectrometry and enrichment techniques. In recent years, with the rapid development of related technologies and the discovery of new histidine phosphatases, researchers have paid more attention to the roles of histidine phosphorylation in tumors. Therefore, we aim to review the roles of histidine kinases and phosphatases in tumor.

蛋白质的可逆磷酸化主要通过蛋白激酶和磷酸酶调控其活性水平，参与细胞生长和代谢等多种功能调节。蛋白质磷酸化可发生在丝氨酸、苏氨酸、酪氨酸、天冬氨酸、谷氨酸、半胱氨酸、组氨酸、赖氨酸和精氨酸等氨基酸残基上，其中蛋白质酪氨酸、丝氨酸和苏氨酸磷酸化在肿瘤发生发展中的作用已经得到了广泛的研究。Boyer等^[1]^首次在线粒体琥珀酰辅酶A合成酶（琥珀酰硫激酶）中发现了组氨酸磷酸化。组氨酸磷酸化是原核细胞信号转导的关键，目前在真核生物中组氨酸磷酸化的研究进展缓慢，主要原因是组氨酸修饰的酸不稳定性和热敏感性，与适用于磷酸丝氨酸、苏氨酸和酪氨酸分析的标准磷酸化蛋白质组方法不兼容。近年来，随着组氨酸磷酸类似物和组氨酸单克隆抗体技术的发展，组氨酸磷酸化的研究取得了一些进展，发现组氨酸磷酸化在细胞中可作为一种可逆修饰调节参与细胞信号传导过程。真核细胞中，组氨酸激酶和磷酸酶通过对含组氨酸的蛋白进行磷酸化调控，参与包括癌症在内的多种疾病的进展。因此，我们将对蛋白质组氨酸激酶和磷酸酶在肿瘤中的研究现状进行综述。

## 1 组氨酸激酶

组氨酸激酶主要存在于双组分信号转导系统中，该系统包括组氨酸激酶（histidine kinase, HK）和相应的调控蛋白。在哺乳动物细胞中发现了多种组氨酸激酶，其中典型的包括组蛋白H4组氨酸激酶（histone H4 histidine kinase, HHK）、二磷酸腺苷酸激酶（nucleoside diphosphate kinase, NDPK）、二组分组氨酸激酶等。

### 1.1 HHK

HHK是最早被描述的哺乳动物组氨酸激酶。最早在再生的大鼠肝细胞核和Walker-256癌肉瘤细胞中发现了HHK^[2, 3]^。组蛋白H4的组氨酸磷酸化发生在肝再生过程中，HHK活性增高与肝脏祖细胞增殖和分化相关，提示HHK可能参与癌症的发生和发展^[4]^。HHK在人肝脏胚胎时期和肝癌组织中活性升高，而在癌旁组织和正常肝脏组织中的活性水平非常低，这种表达模式与其他经典的“肿瘤发育标志物”如甲胎蛋白、丙酮酸激酶同工酶、醛缩酶等的表达模式相似^[5]^，从而提示HHK可能作为潜在的肿瘤发育标志物，以HHK为治疗靶点的抗癌药物开发成为新的可能。

### 1.2 NDPK

NDPK通过ping-pong机制催化γ-磷酸从核苷三磷酸转移到核苷二磷酸，参与高能磷酸组氨酸中间体的形成，从而维持三磷酸腺苷的平衡，在细胞中发挥代谢作用^[6]^。NDPK是由NME（neoplasm metastasis）基因编码的核苷酸代谢酶，因此也称为Nm23（non-metastatic gene 23）蛋白^[7]^。1988年，Steeg等^[8]^首次分离出*Nm23*基因。迄今为止已发现该家族成员有十个不同的蛋白（Nm23-H1-Nm23-H10），根据序列同源性分为两组：H1-H4具有核苷NDPK并有高度相似性（58%-88%），H6-H10蛋白差异明显，仅25%-45%相似度而且表现很少NDPK活性^[9]^。Nm23-H1（NDPK-A或NME1）与Nm23-H2（NDPK-B或NME2）的序列同源性最高（并且它们的基因相邻），研究最为广泛。

NME蛋白作为一种组氨酸激酶，在细胞迁移和侵袭、细胞生长发育、内吞作用和细胞信号转导中发挥重要的作用，例如NME通过G蛋白β亚基1（H266）的组氨酸磷酸化来调节G蛋白的激活；NME通过组氨酸磷酸化钾通道KCa3.1（H358）和钙通道TRPV5（transient receptor potential cation channel subfamily V member 5）（H711）来调节离子通道；NME还通过磷酸化ACLY（ATP citrate lyase）（H760）促进脂肪酸生物合成、SUCLG1（succinate-CoA ligase GDP/ADP-forming subunit alpha）（H299）磷酸化琥珀酰辅酶A和糖酵解重要酶ALDOC（aldolase, fructose-bisphosphate C）在细胞代谢中发挥作用；NME蛋白激酶参与了表皮生长因子受体（epidermal growth factor receptor, EGFR）的活化和内吞作用；NME磷酸化抑制癌细胞的迁移和侵袭等^[6]^。

NME1（neoplasm metastasis 1）在肿瘤的增殖、侵袭、转移以及预后等方面具有重要作用。*NME1*因抑制黑色素瘤转移的能力而闻名，它是目前发现的第一个肿瘤转移抑制基因，其编码的蛋白选择性抑制癌细胞的转移，而对原发肿瘤的生长几乎没有影响^[10]^。在黑色素瘤、乳腺癌、卵巢癌等癌症中均证实NME1的过表达可以抑制肿瘤转移，并增加患者的总生存期^[11]^。肺癌作为最常见的恶性肿瘤之一，吸烟是其主要的危险因素，Wang等^[12]^使用体外致癌模型和生物信息学分析鉴定出肺腺癌中与吸烟相关的基因，明确了NME1与香烟烟雾引起的肺腺癌相关。Kim等^[13]^分析了425例早期非小细胞肺癌患者组织中NME1的表达水平，在39%的样品中发现NME1的表达降低，且NME1表达降低的患者复发率高于NME1未表达降低的患者。罗猛等^[14]^在其建立的稳定沉默*Nm23*-*H1*基因的肺癌细胞株NL9980-99和A549-99中发现，和对照细胞A549相比，*Nm23*-*H1*基因沉默后，细胞的侵袭能力和转移能力增强。分子机制上，Nm23-H1可以抑制转化生长因子-β（transforming growth factor-β, TGF-β）诱导的肺癌细胞A549的上皮细胞间质转化（epithelial-mesenchymal transition, EMT），在肿瘤转移发生的早期发挥抑制肿瘤转移的作用，其中转录因子Snail介导了这一过程^[15]^；同时，Nm23-H1可以负反馈调控白细胞介素-6（Interleukin-6, IL-6）诱导的STAT3 Tyr705位磷酸化^[16]^，其中Nm23-H1的两个酶活性位点（Ser44位和Ser120位）在STAT3（signal transducer and activator of transcription）Tyr705位磷酸化的抑制中起关键作用^[17]^。Nm23-H1相互作用蛋白Tiam1可以作为Src激酶的底物^[18]^，Tiam1是Rho因子GTP酶Rac1的激活因子，Rac1活化后通过刺激钙粘素依赖的细胞和细胞间粘附来抑制肿瘤的侵袭和转移能力。Src激酶可以使Tiam1Tyr384位磷酸化降解，介导Src诱导的EMT过程。Nm23-H1可以与Tiam1直接结合并调控Rac1的活化，因此，Nm23-H1可能通过Src激酶调控Tiam1的磷酸化调控EMT过程。Nm23-H1还与肿瘤化疗和放射治疗的敏感性有关，Wu等^[19]^通过超阵列技术分析非小细胞肺癌A549细胞中培美曲塞处理和未处理细胞之间的基因表达发现，培美曲塞增加了A549细胞中Nm23-H1的表达，*Nm23*-*H1*基因的上调可能成为预测培美曲塞疗效的新的生物标志物。喉癌术后放疗患者肿瘤组织Nm23-H1低表达及其核内表达与肿瘤高复发率及较短的无病生存期相关^[20]^；放射抵抗的鼻咽癌^[21]^和头颈部鳞癌^[22]^肿瘤细胞株较之亲代细胞Nm23-H1的表达增高，并且Nm23-H1出现核内转移。

但是，有研究发现Nm23-H1在某些肿瘤中具有促进转移的作用，并与不良预后有关。Yang等^[23]^通过基因表达综合数据库（gene expression omnibus, GEO）和癌症基因组图谱（The Cancer Genome Atlas, TCGA）发现*Nm23*-*H1*是一种特殊的癌基因，在肝细胞癌中高表达，其表达与肝细胞癌的进展与预后不良有关。在其他组织的癌症如急性髓系白血病、神经母细胞瘤和前列腺癌等^[8, 24]^中也发现Nm23-H1的表达可促进肿瘤转移。Nm23-H1在肿瘤中抑制和促进转移的双重作用可能与NME1在不同组织中的多种生化特性如NDPK活性、组氨酸蛋白激酶活性和3´-5´核酸外切酶活性等的差异有关。

与NME1一样，NME2也可以作为转移调控因子，能在不同的细胞环境下调控肿瘤的发生和发展。NME2蛋白已被鉴定出三种底物，即异源三聚体G蛋白的β亚基（Gβ）、中间电导钾通道KCa3.1和钙传导Trp通道家族成员TrPV5。在这三种蛋白中，特定组氨酸残基的磷酸化对蛋白质功能具有不同的调节作用，参与不同的生理或病理过程。在人非小细胞肺癌H1299细胞中，NME2通过促进原癌基因*c-Myc*表达促进肿瘤的生长^[25]^。NME2缺失的非小细胞肺癌细胞A549的转录本与肺腺癌有显著的相似之处，但表现出更倾向于骨和脑转移^[26]^，晚期肺癌组织中NME2水平显着降低，且具有较高NME2水平的肺癌患者的生存期更长。Thakur等^[27]^通过ChIP-chip研究证实NME2可以作为关键的转录因子，通过与粘着斑蛋白*vinculin*基因的启动子结合，抑制*vinculin*基因转录，从而抑制肺癌细胞的转移能力。NME2可作为转录因子参与基因转录调控，它通过与RNA聚合酶Ⅱ和RNA聚合酶Ⅱ相关蛋白2相互作用，介导RNA聚合酶Ⅱ C末端第5位丝氨酸的磷酸化，促进miR-100和*RIPK1*（receptor interacting serine/threonine kinase 1）、*STARD5*（StAR related lipid transfer domain containing 5）和*LIMS1*（LIM zinc finger domain containing 1）等抗凋亡相关基因的转录，抑制体内外胃癌细胞的凋亡，从而抑制体内肿瘤的生长^[28]^。头颈部鳞状细胞癌、黑色素瘤、胃癌和结直肠癌等肿瘤中也发现NME2高表达可以抑制癌症转移，增加患者生存期。目前对Nm23-H2转移抑制的作用机制了解甚少，Kar等^[29]^报道Nm23-H2通过与端粒酶结合降低体内外的端粒酶活性，其持续表达可改变端粒酶功能和端粒长度，提示Nm23-H2转移抑制作用可能与端粒相互作用相关。在肝细胞癌（hepatoma carcinoma cell, HCC）样本中通过芯片基因微阵列方法检测*Nm23*-*H2*基因的表达水平^[30]^，Nm23-H2蛋白在75%的肝癌组织和肝癌细胞系中过表达，与正常肝、肝硬化和分级肝细胞癌组织比较表明，Nm23-H2的表达与肝癌的恶性程度呈正相关。总之，Nm23-H2不仅仅是转移抑制因子，在不同微环境下，它也可以参与调节肿瘤的发生发展。

Nm23-H3和Nm23-H2具有65%的同源性，可作为核因子κB（nuclear factor kappa-B，NF-κB）的下游靶基因调控粘附蛋白和整合素的表达，还可参与由TLR5（Toll-like receptor 5）诱导的NF-κB的激活，增强下游细胞因子的释放和免疫细胞向肿瘤微环境的募集^[31]^。新近研究发现，NME3通过MyD88调控TLR-5-NF-κB通路；进一步通过*Kaplan Meier*生存分析发现，在乳腺癌、肺癌、卵巢癌和胃癌中，NME3表达与TLR5表达高度相关，且NME3高表达可提高乳腺癌、肺癌和卵巢癌患者的总生存率，但在胃癌患者中则相反^[32]^。这些研究提示NME3可作为TLR5下游信号发挥促炎作用，进而可能增强癌症的免疫治疗^[32]^。

最近在肺癌中研究^[33]^发现，NME4（Nm23-H4）在肺癌组织和多种肺癌细胞中存在过表达，通过慢病毒shRNA干扰NME4的基因表达后，细胞周期阻滞在G1期，细胞增殖和克隆形成能力也受到抑制，从而提示NME4可能作为一种新型的促癌因子，能够通过克服细胞周期停滞和促进增殖来促进非小细胞肺癌的进展。Nm23-H6，特别是Nm23-H4和Nm23-H7可能参与结肠癌和胃癌的发展，然而，目前的研究无法证明它们对肿瘤进展或转移有贡献。Nm23-H5-Nm23-H10很少表现出NDPK活性，目前对它们的研究开展甚少，在肿瘤中发挥的作用有待深入研究。

### 1.3 二组分组氨酸激酶

二组分组氨酸激酶是研究最多的组氨酸激酶，是存在于细菌、真菌和植物中的双组分信号激酶。二组分系统（tow-component system, TCS），也称为组氨酸-天冬氨酰磷（histidine aspartic phosphorus, HAP）系统，该系统由HK和相应的调控蛋白组成。细菌利用TCS快速感应并响应各种环境信号，因此TCS在细菌病理毒理中具有重要意义，被认为是抗菌药物研发的靶标^[34]^。Attwood等^[35]^指出来源于高等真核生物的一些蛋白，其与双组分组氨酸激酶系统的蛋白质成分具有序列和结构上的相似性。因此，在高等真核生物中可能存在完整的二组分组氨酸激酶系统但尚未被发现，它们在真核生物中的作用也有待研究。

## 2 组氨酸磷酸酶

组氨酸磷酸化可以被组氨酸磷酸酶逆转，目前已知的组氨酸磷酸酶包括磷酸组氨酸磷酸酶1（phosphohistidine phosphatase 1, PHPT1）、磷酸甘油变构酶5（phosphoglycerate mutase 5, PGAM5）和磷酸组氨酸无机焦磷酸酶（phosphohistidine inorganic pyrophosphate phosphatase, LHPP）。在细胞信号转导过程中组氨酸磷酸酶起着至关重要的作用，它们的表达和功能失调在肿瘤的发生发展过程中具有重要作用。

### 2.1 PHPT1

人磷酸组氨酸酸酶1是脊椎动物中发现的第一个蛋白组氨酸磷酸酶。目前，对它的生物学功能还知之甚少。Ek等^[36]^证明PHPT1能使磷酸化组蛋白H1和聚赖氨酸去磷酸化，这一发现显示了该酶具有更广泛的特异性，并可能成为研究蛋白磷酸化有价值的工具。通过免疫组化检测146例肺癌组织和30例癌旁组织中PHPT1蛋白的表达水平，结果表明肺癌组织中PHPT1的表达明显高于正常组织^[37]^；同时发现PHPT1与肿瘤侵袭有关，体外细胞迁移和侵袭实验结果发现PHPT1下调可显著抑制肺癌细胞体内转移，进一步通过蛋白质组学分析发现PHPT1还可能通过调控肌动蛋白介导的细胞骨架重排调控肺癌细胞转移和侵袭能力^[38]^。Shen等^[39]^在122例肾透明细胞癌组织标本中发现PHPT1在肾癌组织的近端小管上皮和细胞核中均有表达，且PHPT1高表达与肿瘤大小、Fuhrman核分级和晚期肿瘤淋巴结转移相关，与PHPT1高表达的患者相比，PHPT1低表达的患者的总生存期增加。病毒介导的RNA干扰技术敲除肝癌细胞*PHPT1*基因，细胞增殖受到明显抑制，细胞凋亡明显增加^[40]^。以上研究提示，PHPT1可通过调控肿瘤细胞的增殖和细胞凋亡在肿瘤发生发展中起作用，可能是肿瘤治疗的潜在靶点。

### 2.2 PGAM5

PGAM5是磷酸甘油酸变位酶超家族成员之一，是定位于线粒体的丝氨酸/苏氨酸蛋白磷酸酶。Panda等^[41]^发现PGAM5还是一种组氨酸磷酸酶，可使NDPK-B上的组氨酸118位（H118）特异性去磷酸化，从而抑制NDPK-B的磷酸化和KCa3.1的活化。*PGAM5*突变发生在多种肿瘤中，这些突变可能会改变PGAM5在线粒体动力学和程序性细胞死亡中的作用，并促进肿瘤的发展^[42]^。Wang等^[43]^报道PGAM5通过调节线粒体的裂变和坏死来诱导人类结肠癌细胞和宫颈癌细胞的死亡。Cheng等^[44]^证明PGAM5在肝癌中的表达显著高于相应的癌旁肝组织，此外，PGAM5低表达抑制了肿瘤的生长并增加了肝癌细胞对5-氟尿嘧啶的敏感性。Ng等^[45]^通过免疫组织化学发现PGAM5在非小细胞肺癌组织中表达，但在正常上皮中不表达，因此其可能在气道上皮细胞的恶变过程中起一定作用，且PGAM5在肺癌中的表达与患者的存活率有关，提示PGAM5可参与多种肿瘤的进展。

### 2.3 磷酸赖氨酸磷酸组氨酸无机焦磷酸磷酸酶（phospholysine phosphohistidine inorganic pyrophosphate phosphatas, LHPP）

Hiraishi等^[46]^首次在牛肝中分离出一种新型的56 kDa无机焦磷酸酶，称为LHPP。在肝脏特异性缺失PTEN（phosphatase and tensin homolog）和TSC1（TSC complex subunit 1）的L-dKO肝癌小鼠模型中，Hindupur等^[47]^发现小鼠肝癌中的整体组氨酸磷酸化显著上调，其中组氨酸磷酸化激酶NME1和NME2上调，LHPP下调，并进一步证实LHPP是一种组氨酸磷酸酶，通过体外细胞和体内动物实验发现LHPP过表达可以抑制癌细胞增殖、降低肿瘤负荷并减缓肝功能降低，因而提示LHPP是一种蛋白组氨酸磷酸酶和肿瘤抑制因子，解除组氨酸磷酸化是致癌的。但LHPP在人肝癌中的作用和机制仍很不清楚。有研究人员利用生物信息学方法预测和分析LHPP的结构和功能时，发现LHPP属于酸性、稳定、疏水、非分泌型、无跨膜区的蛋白，主要存在于细胞质，线粒体、细胞核和内质网也可动态存在^[48]^。LHPP的组织表达特异性不强，在多数组织中均有表达，但在脑、肾、肝、甲状腺等组织中最为丰富。新近研究也表明LHPP与癌症的发生发展密切相关。LHPP低表达导致蛋白组氨酸磷酸化水平升高，细胞发生增殖失控，导致肿瘤生长、扩散，而LHPP过表达可以抑制肿瘤形成。Liao等^[49]^发现LHPP在人肝癌组织中的表达水平与细胞周期演进、有丝分裂纺锤体形成、G_2_期-M期检查点和肿瘤转移呈负相关，LHPP在肝癌细胞中表达下调，过表达LHPP可抑制肝癌细胞的生长和转移，分子机制上，LHPP可降低CCNB1（Cyclin B1）、PKM2（pyruvate kinase isozyme type M2）、基质金属蛋白酶7（matrix metallopeptidase 7, MMP7）和基质金属蛋白酶9（matrix metallopeptidase 9, MMP9）等促癌或转移促进基因的表达水平。非小细胞肺癌的发病率和死亡率居世界首位，顺铂作为非小细胞肺癌常用的化疗药物之一，其耐药性是肺癌治疗失败的主要原因之一，Yang等^[50]^研究揭示Gas5/miR-217/LHPP通路可降低非小细胞肺癌顺铂耐药，提示LHPP可能成为非小细胞肺癌顺铂耐药的潜在治疗靶点。在大肠癌、肾细胞癌、乳头状甲状腺癌、胰腺癌、膀胱癌和宫颈癌等多种肿瘤中均发现LHPP过表达可以抑制上述肿瘤细胞的增殖和侵袭。LHPP的表达水平与肿瘤的分期和进展、患者的生存期相关，提示LHPP可作为肿瘤诊断和预后的标志物，为肿瘤治疗提供新的靶点和思路。

## 3 结语

综上所述，组氨酸激酶和组氨酸磷酸酶通过调节细胞内组氨酸磷酸化水平，在肿瘤的增殖、侵袭、转移以及不良预后等方面具有重要作用。HHK和NDPK，尤其是NME蛋白，作为组氨酸激酶通过抑制或者促进肿瘤转移，在肿瘤进展中起重要作用，特别是Nm23-H1在非小细胞肺癌转移的中的抑制作用，可作为肺癌转移潜能的生物学指标。PHPT1、PGAM5和LHPP作为组氨酸磷酸酶也在多种肿瘤的增殖和转移中具有重要的调控作用。组氨酸激酶和磷酸酶作为调控细胞内组氨酸磷酸化水平的关键因子，到目前为止，对二者尤其是组氨酸磷酸酶在肿瘤中的作用和分子机制研究尚少，需进一步探索，同时仍需发现更多未知的蛋白质激酶和磷酸酶，为临床上肿瘤的诊断和治疗提供更多新的思路和靶点。
